# Perspiration

**Published:** 1856-11

**Authors:** 


### PERSPIRATION,

Is the transfusion of water from the interior of the body, through the skin, to without us. This transfused fluid is not pure water, it is saltish to the taste, and it conveys, is the carrier of, a large amount of various impurities out of the body ; it is one of the scavengers of the human frame. If the passage ways, the hose-pipes, through which the perspiration is conducted, are closed, these impurities are retained, are remixed with the blood and the whole mass of it becomes impure from that cause within two minutes and a half; and every two minutes and a half the impurity is more and more concentrated, and so rapidly does this corrupting process go on, and so deleterious are its effects, that if the whole of them are kept closed, by any gummy substance, or we are completely enveloped with an India rubber garment, we would die in a few hours..

*Moderate exercise* keeps these passages open, hence those persons who are moderately exercising all day, whether in or out of doors, are the longest lived, the world over. This moderate exercise is to the body, what a fire engine or a common pump is in practical life, it keeps the fluid passing along, and as it passes, washes us clean of all impurities.

A quart of water, laden with concentrated impurities, passes through the skin of a healthy person every twenty four hours, hence the necessity of keeping these sluices of the system always in operation, by moderate exercise, and their extensive openings free, by the strictest habits of thorough personal cleanliness.

This one idea, of keeping the pores of the skin steadily open by means of habitual moderate exercise and strict personal cleanliness, would, if generally practiced, contribute more to human happiness than tons of physic or millions of money.

